# Harvesting fodder trees in montane forests in Kenya: species, techniques used and impacts

**DOI:** 10.1007/s11056-018-9632-x

**Published:** 2018-02-24

**Authors:** Aida Cuni-Sanchez, Marion Pfeifer, Rob Marchant, Patrícia V. Pompeu, Neil D. Burgess

**Affiliations:** 1 Center for Macroecology, Evolution and Climate, University of Copenhagen, Universitetsparken 15, 2100 Copenhagen, Denmark; 2 Environment Department, University of York, Heslington, York, YO10 5DD UK; 3 School of Biology, Newcastle University, Ridley Building 2, Newcastle upon Tyne, NE1 7RU UK; 4 Department of Forestry, Federal University of Lavras, PO Box 3037, Lavras, Brazil; 5 United Nations Environment Programme World Conservation Monitoring Center, 219 Huntingdon Road, Cambridge, UK

**Keywords:** Carbon stocks, East Africa, Forest conservation, Population structure

## Abstract

There has been an increasing interest in fodder trees and their potential to help the rural poor. However, few studies have addressed the ecological impacts of fodder tree harvesting. We investigated the species harvested and the techniques used, and the effects of fodder harvesting on (1) species’ populations and (2) forest carbon stocks in three montane forests in Kenya. Focus-group discussions were organized in 36 villages to determine which species were harvested and with which techniques. Field observations were made on vegetation plots: stem diameter, tree height, species and extent of harvest were recorded. Carbon stocks were calculated using an allometric equation with (1) observed height of harvested trees, and (2) potential height estimated with a power model, and results were compared. Eight tree species were commonly harvested for fodder using different techniques (some branches, main stem, most branches except stem apex). Fodder harvesting (together with other uses for some species) negatively affected one species populations (*Olea europaea*), it did not negatively affect four (*Drypetes gerrardii, Gymnosporia heterophylla, Pavetta gardeniifolia, Xymalos monospora*), and more information is needed for three species (*Olea capensis*, *Prunus africana, Rinorea convallarioides*). Fodder harvesting did not significantly reduce forest carbon stocks, suggesting that local communities could continue using these fodder trees if a carbon project is established. Among the fodder species studied, *X. monospor*a could be used in reforestation programs, as it has multiple uses and can withstand severe pruning. Although our study is only a snapshot, it is a baseline which can be used to monitor changes in fodder harvesting and its impacts related to increasing droughts in northern Kenya and increasing human populations.

## Introduction

Fodder tree harvesting is undertaken in a wide range of systems in Africa (Franzel et al. 2014). For instance, in the parklands of West Africa, more than 70 tree species are used for this purpose (Ouedrago et al. 2000). As most tree leaves have high nitrogen content compared with grasses, they augment the low nutritional value of crop residues and natural pastures which are the base for livestock production in Africa (Mekoya 2008). Fodder trees are also used to meet production shortages in pasture in times of extreme climatic conditions such as droughts (Franzel et al. 2014; Balehegn et al. 2015).

There has been an increasing interest in fodder trees and their potential to help the rural poor as they have a positive effect on milk production in livestock and hence local income generation (Chakeredza et al. 2007; Place et al. 2009). Several studies from across Africa have documented the positive impacts of fodder trees on human livelihoods (e.g. Franzel 2004 in Kenya, Tanzania and Zambia; Wambugu et al. 2006 across East Africa; Toth et al. 2017 in Malawi). Incorporating this type of trees into agro-silvopastoral systems can also contribute to environmental rehabilitation, e.g. improving soil fertility (Moreno and Obrador 2007). In particular, their inclusion in agro-silvopastoral systems has recently gained prominence as a sustainable and climate resilient livestock production system (Mosquera-Losada et al. 2011; Balehegn 2017).

Despite their ecological and economic potential, limited research attention has been given to indigenous fodder trees, compared to exotic species (Le Houèrou 2000). Most indigenous fodder trees are multipurpose and provide multiple benefits and services (e.g. food, fibre, medicine, fire-wood) (Balehegn and Eniang 2009). Indigenous fodder species are often preferred by local farmers over exotic species for being of low cost (seeds or seedlings can be collected the wild), available and accessible to local communities, adapted to local environmental conditions (requiring little or no management input), and resistant to diseases and parasites (Le Houèrou 2000; Bayala et al. 2014).

One aspect of indigenous fodder trees which remains understudied is the impacts on plant populations when harvesting from the wild. Wild plant extraction affects the rates of survival, growth and reproduction of harvested individuals (Ticktin 2004). Delays in growth, fruit and seed production, and subsequent regeneration or sprouting, are typical because plant resources (e.g. nutrients) are relocated from reproduction to healing the ‘wounds’ created from harvesting (Hall and Bawa 1993). These changes can in turn affect the structure and dynamics of harvested plant populations (Ticktin 2004). Slow-growing old-growth tree forest species that occur in low densities and multipurpose tree species are particularly vulnerable to overharvesting (Peters 1996; Gaoue and Ticktin 2007; Houehanou et al. 2011).

Harvesting wild plants may also affect forest structure and composition (Ticktin 2004), and forest carbon stocks. While logging is known to affect forest carbon stocks (Nepstad et al. 1999), it has been reported that the removal of small stems for firewood does not significantly influence forest carbon stocks (Lung and Espira 2015), because large stems contribute towards most forest carbon (Marshall et al. 2012). If the shrubs or trees harvested for fodder are small individuals, it is also likely that their removal does not affect forest carbon stocks. Yet, to our knowledge, no study has empirically assessed if this is the case. Considering the increasing interest in carbon finance mechanisms for montane forests in East Africa, this is an important research question. In Africa, many people depend on both lowland and montane forests for their livelihoods (Fisher et al. 2009; 2011).

Before indigenous fodder tree harvesting from the wild is further promoted or limited (e.g. under the establishment of a carbon project) we need to understand which species are being used, how are they harvested and the impacts of this harvesting. As highlighted by Swaine et al. (1990), our current understanding about tropical forest dynamics, much of which has been developed in wet lowland forests, is not always applicable to the tropical forests found at higher elevations in East Africa. The main objectives of this study, focused on three montane forests in Kenya, were to assess: (1) which fodder species and techniques were used, (2) if fodder tree harvesting negatively affected the species populations’ involved, and (3) if it significantly reduced forest live tree carbon stocks. We then discuss the implications of the findings for forest management, including reforestation and carbon projects.

## Materials and methods

### Study area

This study focused on three isolated forested mountains in northern Kenya: Mt Nyiro (2752 m), Mt Kulal (2285 m) and Mt Marsabit (1707 m) (Fig. 3 Appendix). Most of northern Kenya is characterized by lowland terrain classified as very arid with annual rainfall between 150 and 350 mm (zone VII, Sombroek et al. 1982). However, the mountains we studied are much wetter and cooler, with annual rainfall between 800 and 1400 mm (semi-humid area, zone III Sombroek et al. 1982). Rainfall is concentrated in two wet seasons, from March to May and from October to December, but there is great inter-annual variation with some years having one or no rainy season.

All three mountains support similar vegetation types. From lower to higher altitudes, forests can be divided into: (1) ‘dry montane forest’ (*Croton megalocarpus* Hutch.- *Olea europaea* subsp. *cuspidata* (Wall. and G.Don) Cif. forest association in Mt Marsabit or *O. europaea*-*Juniperus procera* Hochst. ex Endl. forest association in Mt Kulal and Mt Nyiro), (2) ‘mixed species forest’ (with abundant *Cassipourea malosana* (Baker) Alston and *Olea capensis* L. in all mountains) and (3) ‘elfin-like forests’ (short trees with twisted stems covered with mosses and lichens) (Bussmann 2002). Because of its lower altitude, no elfin-like forests are found in Mt Marsabit. Some large grassy clearings are also found on top of Mt Kulal and Mt Nyiro.

Mt Nyiro, Mt Kulal and Mt Marsabit are part of the Eastern Afromontane Biodiversity Hotspot (Bird Life International 2012). Mt Marsabit is a national park, Mt Nyiro is a forest reserve and Mt Kulal is a community forest. Management guidelines for Mt Marsabit allow free access for non-timber products, but grazing and fodder tree harvesting are restricted to drought events and firewood collection is illegal. Access to Mt Nyiro and Mt Kulal forests is not restricted. Apart from being a dry season grazing ground, they are an important source of firewood and other non-timber forest products (NTFPs) (Cuni-Sanchez et al. 2016). Mt Marsabit forest is an important elephant habitat (Ngene et al. 2009), but there are no elephants on Mt Kulal or Mt Nyiro. Commercial logging never occurred on these mountains because of the steep terrain and remoteness of the area. Mt Nyiro and Mt Kulal are only populated by Samburu pastoralists while different ethnic groups inhabit Mt Marsabit (Fig. 3 Appendix). Herd composition is similar across mountains; people prefer cows over goats for cultural reasons, and sheep are rare. Herd size depends upon household income (i.e., richer people owning more cows) and does not depend upon mountain or ethnic group (unpublished questionnaires).

### Patterns of fodder harvesting

Focus-group discussions (FGDs) were organized in twelve permanent villages located around each of the three mountains (3 × 12 = 36, Fig. 3 Appendix) between October and December 2015. This encompassed all major permanent villages in each mountain. Each FGD involved 5–10 male elders including the village chief, as it is a custom in the area. Participants were selected on a voluntary basis after they were explained the purpose of our study. FGDs were facilitated and translated by a person of the same ethnicity of FGD we were working with. All participants in this study were male. As males are generally in charge of herd feeding and because of time constraints, we did not organize a female FGD in each village (note that in the study area females do not talk openly in front of males due to cultural norms).

FGDs centered on five topics: the most important fodder trees, their uses, current harvesting techniques, time of the year when fodder tree harvesting takes place and perceptions of the status of their populations (increasing/steady/declining). All plant species mentioned in a FGD were collected for identification and verification of their local name at the Herbarium of University of Nairobi. Field observations were also made in each forest to determine if the plants mentioned in FGDs were present and how they were being collected. Species presence in a mountain and their conservation status were also checked with the literature (e.g. Beentje 1995). In Mt Kulal the Samburu name ‘sagumai’ was used for two species: *Gymnosporia heterophylla* (Eckl. and Zeyh.) Loes. (synonym = *Maytenus heterophylla*) and *Maytenus undata* (Thunb.) Blakelock. To simplify, *Gymnosporia heterophylla* is used throughout the text, as this species was more abundant.

Population density and structure were only assessed for the most important fodder species, i.e. those mentioned in a larger number of FGDs in each mountain. *Prunus africana* (Hook.f.) Kalkman only used for fodder in Mt Nyrio, was also studied because it is listed as vulnerable by the IUCN Red List of Threatened Species. The bark of this species is traded internationally for its use against benign prostatic hyperplasia (Stewart 2003).

### Population density and structure

We studied species’ density and population structure, as population structure assessments are known to be cost-effective means of providing useful data on the impact of harvesting and other disturbances, contributing to the development of effective management strategies (Botha et al. 2002). Three permanent 0.2 ha rectangular plots (20 × 100 m) were established perpendicular to the main slope in each forest type encountered in each mountain (n = 9 in Mt Kulal, n = 9 in Mt Nyiro but n = 6 in Mt Marsabit, as there is no elfin forest in this mountain). Plots were located < 100 m from footpaths, which is likely to be representative of an average forest utilization area. Humans access most parts of these forests to collect medicinal plants, travel between villages and allow their cows to reach water points, etc. The only parts of these forests with little human presence and no plant harvesting are very steep rocky areas. Fewer larger rectangular plots were preferred over many small plots because these are better for tree growth monitoring (e.g. Gaoue and Ticktin 2007), which was also an objective of our research for the future (in 5 years).

**Fig. 1:**
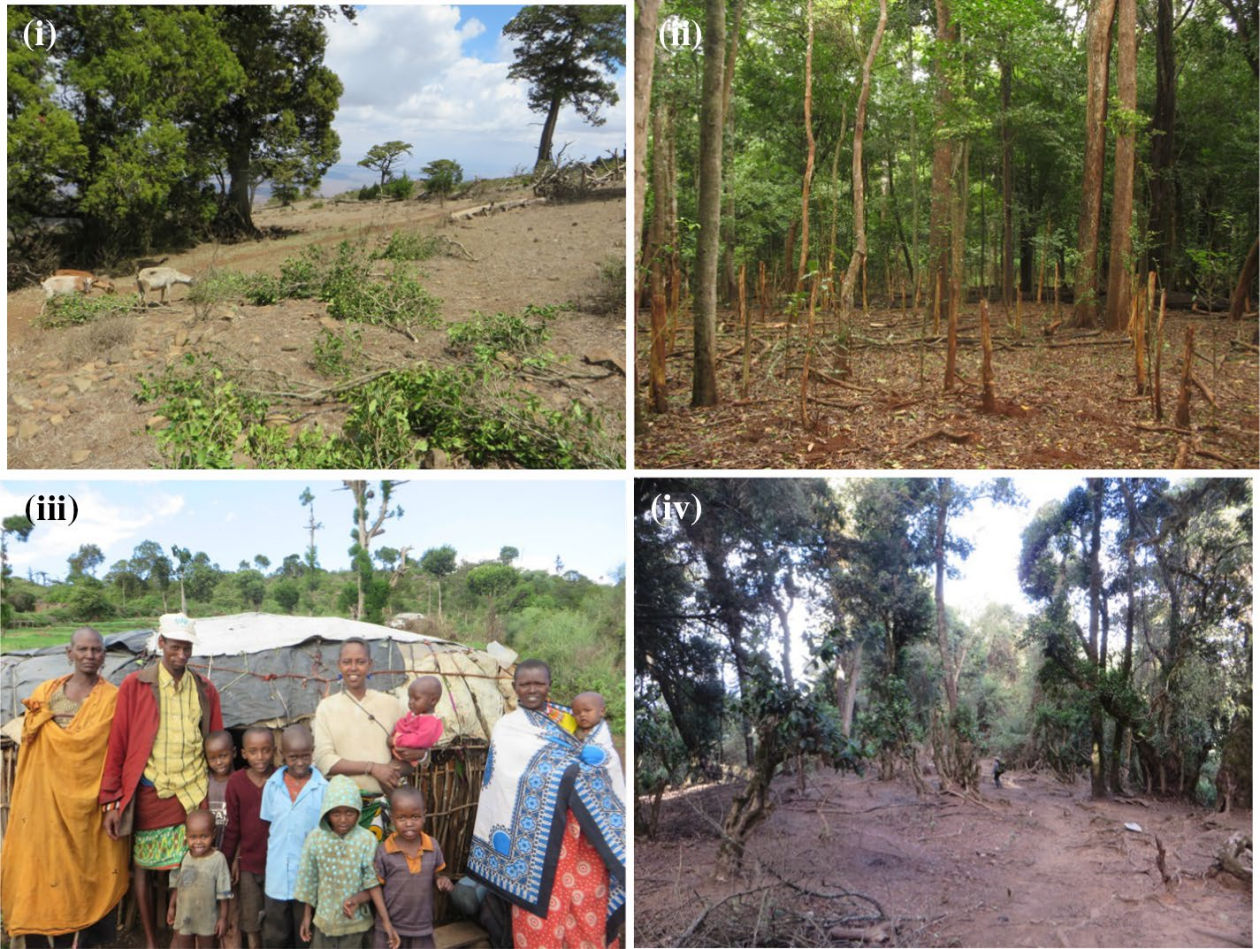
From top to bottom clockwise: (**i**) fodder tree harvesting of *Pavetta gardeniifolia* in Mt Kulal (small branches cut, note how dry is the grassland on top of the mountain during the dry season), (**ii**) fodder tree harvesting of understory *Rinorea convallarioides* in Mt Marsabit (note number of dead yellowish stems of this species), (**iii**) fodder tree harvesting of canopy *Olea capensis* in Mt Kulal (see few branches unharvested on top trees, background of the picture, Samburu pastoralist family in front); and (**iv**) fodder tree harvesting of understory *Xymalos monospora* in Mt Nyiro (note stems pruned > 6 m, right side picture)

In each plot, tree diameter at 1.3 m along the stem from the ground (or above buttresses if present) and tree height (measured using a handheld laser Nikon Forestry Pro) of each tree ≥ 10 cm diameter were recorded following RAINFOR protocols (www.rainfor.org), and stems were identified to species where possible. Standing dead trees ≥ 10 cm diameter were also recorded. Stem density (number trees ha^−1^) included all trees ≥ 10 cm diameter. In the plots, we assessed trees ≥ 10 cm diameter because locals mentioned not harvesting branches/bark from small trees so that ‘*they can grow*’ (participant comments during FGDs). Standing dead trees ≥ 10 cm diameter of *Rinorea convallarioides* Eyles, which have a characteristic yellowish color (Fig. 1) and were particularly abundant in Mt Marsabit, were used to compute dead stems ha^−1^ for this species. For the other species, standing dead stems were found to be < 1 stem ha^−1^ and are not reported in Table 1.

**Table 1:** General information about the species studied, mountains where they are found, preference as fodder tree (first/second/third best species for fodder), other uses, perception of declining populations, forest types where it can be found, stem density, percentage of severely pruned trees, dead trees and size-class distribution (SCD) slopes

Species	Mountain	Fodder tree	Uses	Perception of declining populations	Forest types	Stem density (stems ha^−1^)	Severely pruned trees (%)	Dead trees (stems ha^−1^)	SCD slopes
*Olea capensis*	Mt Nyiro	First	Fodder, firewood	Yes	Dry, mixed, elfin	10–130	11		− 1.27
	Mt Kulal	First	Fodder, firewood, poles	No	Dry, mixed, elfin	10–110	2		− 1.47
	Mt Marsabit	Third	Fodder only	No	Mixed	10–115	0		− 1.97
*Olea europaea*	Mt Nyiro	Third	Fodder, firewood	No	Dry	95–125	0		− 1.40
	Mt Kulal		Firewood, poles, (fodder)	No	Dry	30^a^	0		na
	Mt Marsabit	Third	Fodder, firewood, poles, food, medicine	Yes	Dry	10–20	0		0.01
*Xymalos monospora*	Mt Nyiro	Second	Fodder, poles	No	Mixed, elfin	145–285	70		− 2.92
	Mt Kulal			No	Mixed, elfin	15–105	0		− 1.87
*Prunus africana*	Mt Nyiro		Fodder, firewood	No	Mixed, elfin	10–20	0		− 0.40
	Mt Kulal			No	Elfin	30–95	0		− 1.43
*Pavetta gardeniifolia*	Mt Kulal	Third	Fodder only	No	Dry, mixed, elfin	15–135	0		− 3.75
*Gymnosporia heterophylla*	Mt Kulal	Second	Fodder only	No	Elfin	25–45	0		na
*Rinorea convallarioides*	Mt Marsabit	Second	Fodder only	Yes	Mixed	50–125	2	20–85	− 3.17
*Drypetes gerrardii*	Mt Marsabit	First	Fodder, firewood, poles	No	Dry, mixed	70–400	5		na

^a^Refers to only one plot sampled had this species, na: non-available

*na* non-available

To allow for visual comparisons of population structure, size-class distributions (SCDs) were constructed and displayed graphically. Size-classes were determined as 5 cm increments in tree diameter for all species except for *R. convallarioides* (for which 2 cm classes were used) as all stems of this species were < 20 cm in diameter. SCD slope was also used as indicators of population trends. A least-squares linear regression was performed on the data of the SCD, with size-class midpoint (ln transformed) as the independent variable and the average number of individuals per size-class (ln (Ni + 1)) as the dependent variable, as described in Lykke (1998) and Condit et al. (1998). In general, negative SCD slopes indicate good recruitment, flat slopes indicate equal numbers of individuals in small and large size-classes and positive slopes indicate poor recruitment (Obiri et al. 2002).

The extent of fodder tree harvest was assessed on a categorical scale with two classes: severely pruned (top of main stem cut) and non-severely pruned (top of main stem not cut). The category ‘non-severely pruned’ included trees with partial pruning (cutting of some branches) and trees with no pruning, as it was difficult to distinguish one from another especially for tall individuals and individuals with large crowns. For each species, the percentage of trees being severely pruned was computed. After sampling the plots in Mt Marsabit, we realized that only six individuals of *Olea europaea* had been sampled, which did not allow for the construction of SCD. As this species was mentioned as being in decline by local communities (see results), we identified two locations in the eastern part of the forest where this species was relatively abundant, we sampled 125 individuals in each and we computed SCD with this data.

Fodder tree harvesting might not be the only factor affecting the species’ populations studied. Wood harvesting for firewood, medicinal plant collection, illegal logging, grazing of seedlings by wild or domestic animals, and droughts (among others), might also be affecting these tree species’ populations. FGDs provided insights into human uses (other than fodder). Because of the remoteness of these mountains, no fodder species studied is commercially traded outside each mountain (FGDs comments and personal observations). For instance, no *Prunus africana* bark reaches Nairobi herbal market (Delbanco et al. 2017). With regard to wild animals, elephants do not target the fodder species we studied (Marsabit NP ranger comment, 2015). Only two individuals of *Drypetes gerrardii* Hutch. had been felled by elephants in our plots in Mt Marsabit.

We were unable to assess seedling and saplings in our plots because of the difficulty in finding them during the period in which fieldwork took place (after a long dry season). Although seedling browsing by goats or cows can affect seedling presence, we did not see seedlings even in steep slopes with no browsing. Lack of seedlings might be related to reduced fog presence in these mountains (see Cuni-Sanchez et al. in press).

### Forest carbon stocks

Above ground live tree carbon stocks (AGC) were calculated: (1) including severely pruned trees (named AGC pruned) and (2) including the potential height of these severely pruned trees had they not been pruned (named AGC non-pruned). First, the above ground biomass (AGB) of each tree in the plot was estimated using the Chave et al. (2014) equation including diameter, wood mass density and tree height. The best taxonomic match wood mass density of each stem was extracted from a global database (Chave et al. 2009; Zanne et al. 2009) following Lewis et al. (2013). Then, AGB was converted to AGC using the carbon fraction 0.47 (Martin and Thomas 2011). Although carbon fraction varies among species (e.g. Weber et al. 2017) we did not consider this variation as it was beyond the scope of this study.

For AGC pruned, tree height measured in the field of the severely pruned trees was used. For AGC non-pruned, the potential heights of these stems were calculated as follows. First, the relationship between tree diameter and height was established using a Power-model (see Feldpausch et al. 2011), for each forest type in each mountain separately (as climate and population origin can shape height-diameter allometry e.g. Vizcaino-Palomar et al. 2017). Then, these relationships were used to estimate tree height for the trees with severely pruned stems. R statistical software R v3.2.1 and RStudio v.0.99.447 were used for all statistical analyses (R Core Team 2014). Differences in AGC were tested using paired t-tests.

## Results

### Patterns of fodder harvesting

The preferred species for fodder were *Olea capensis, O. europaea* and *Xymalos monospora* (Harv.) Baill. in Mt Nyiro, *O. capensis, Pavetta gardeniifolia* Hochst. ex A.Rich. and *Gymnosporia heterophylla* in Mt Kulal, and *Drypetes gerrardii, Rinorea convallarioides, O. europaea* and *O. capensis* in Mt Marsabit (Table 1). Most species mentioned were multipurpose, with *O. europaea* having several uses in Mt Marsabit (Table 1). Interestingly, *X. monospora* and *Prunus africana* were used for fodder in Mt Nyiro but not in Mt Kulal, despite these two species being abundant in Mt Kulal and both mountains being populated by the same ethnic group.

In Mt Nyiro local populations perceived that *O. capensis* populations were declining (Table 1), as some adult trees were dying and there was little natural regeneration (FGD participants’ comments). Participants mentioned that ‘*O. capensis dries up when it is cut too much while X. monospora can be pruned more intensively as it resprourts easily*’. In Mt Marsabit it was perceived that *O. europaea* and *R. convallarioides* populations were declining (Table 1), as some adult trees were dying and there was little natural regeneration for *O. europaea* (FGD participants’ comments). Some participants mentioned that in Mt Marsabit *‘most tree species’ populations are declining because of overexploitation but the situation is worse for O. europaea as it has many uses*’. With regard to *R. convallarioides*, it was mentioned that ‘*few individuals of this species sprout and now there are many droughts and more people so this species has no time to recover* [after drought events during which many stems are cut]’. No species was perceived as declining in Mt Kulal.

Fodder tree harvesting was carried out in different ways using different techniques depending on the species: cutting some branches, cutting the main stem, or cutting most branches except the stem apex.(Figure 1). Some branches: for *O. europaea* and *P. africana*, which tend to be large individuals, male herders climb them to cut a few branches (male as this requires physical strength, not because it is taboo for women). For *Gymnosporia heterophylla, Pavetta gardeniifolia* and *Drypetes gerrardii,* which tend to be smaller individuals, both male and female herders cut a few branches. Main stem: male/female herders cut whole stems of two species: *R. convallarioides* and *X. monospora.* For *R. convallarioides,* stems are generally cut about 1.5 m from the ground. For *X. monospora* trees, which are often multi-stemmed, stems are generally cut about 5 m from the ground. Most branches except stem apex: for *O. capensis,* carried out by males (as this requires physical strength). For this species, trees which are pruned at 75–99% of their crown cover still possess a few top branches that have leaves (Fig. 1). Leaving these top branches is a traditional practice said to allow the tree to grow and reproduce. With regard to time of the year when fodder tree harvesting takes place, clear differences were observed between mountains. In Mt Kulal and Mt Nyiro it was mentioned that fodder tree harvesting was only necessary at the end of the dry season of some years (drought years) as the large grassy clearings on top of Mt Kulal and Mt Nyiro provided enough pasture. In these two mountains most families bring their animals to the lowlands during the rainy season so that grass can regenerate on the mountains, and the grassy clearings on top are only used during the dry season. In Mt Marsabit it was also mentioned that fodder tree harvesting was only necessary during drought events, during which *R. convallarioides* trees were cut in great numbers. However, field observations and further discussions with some herders indicated that fodder tree harvesting in Mt Marsabit was carried out every dry season (two dry seasons per year) as there was no pasture left outside the forest during the dry seasons. During these dry seasons the only sources of water (wells) are located inside the forest and herders cut branches of trees on the way to these wells. Some villages have to cross > 2 km of forest to reach the well they use, offering a great opportunity for fodder tree harvesting along the path. This bi-annual fodder tree harvesting is of questionable legitimacy and local people know this, which explains why this was not discussed openly in the FGDs.

### Species’ populations

In all mountains studied, *O. capensis* was found at variable densities in all forest types, while *O. europaea* was only found in dry forests and was more abundant in Mt Nyiro (Table 1). *P. africana* was more abundant in Mt Kulal (where it is not used for fodder) than in Mt Nyiro, (where it is used for fodder, see Table 1). In contrast, *X. monospora* was more abundant in Mt Nyiro (where it is used for fodder) than in Mt Kulal (where it is not used for fodder, see Table 1). The other species assessed had variable densities, with *D. gerrardii* being highly abundant in Mt Marsabit (Table 1).

Fodder tree harvest pressure was particularly high for *X. monospora* in Mt Nyiro, with 70% of trees being severely pruned (Table 1). Severe pruning affected all diameter classes (Fig. 4 Appendix). With regard to other species, 11% and 2% of *O. capensis* trees were severely pruned in Mt Nyiro and Mt Kulal (respectively), while 5% and 2% of *D. gerrardi* and *R. convallarioides* (respectively) trees were severely pruned in Mt Marsabit (Table 1). In Mt Marsabit a high density of standing dead trees of *R. convallarioides* was observed (Table 1), which was related to overharvesting as reported in FGDs.

**Fig. 2:**
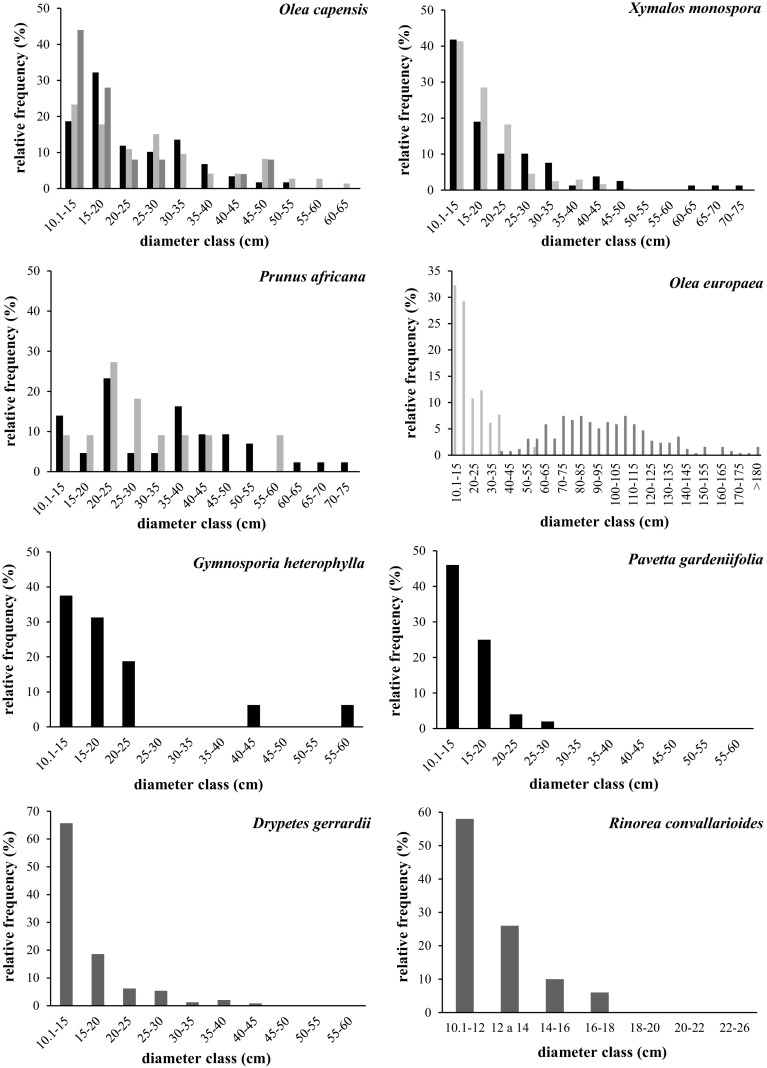
Size class distribution of *Olea capensis, Xymalos monospora, Prunus africana, O. europaea, Gymnosporia heterophylla, Pavetta gardeniifolia, Drypetes gerrardii,* and *Rinorea convallarioides* for Mt Kulal (black), Mt Nyiro (light grey) and Mt Marsabit (dark grey). Note that if a species was not found in a mountain it is not included in the figure (with exception of *O. europaea* for which only 6 individuals were sampled in Mt Kulal and its size class distribution could not be computed). In total we sampled the following number of individuals (K = Kulal, N = Nyiro, M = Marsabit): Olea capensis (K = 59, N = 73, M = 50), Xymalos monospora, (K = 79, N = 242), Prunus africana (K = 43, N = 44), O. europaea (N = 65, M = 250), Gymnosporia heterophylla (K = 32), Pavetta gardeniifolia (K = 77), Drypetes gerrardii (M = 242) and Rinorea convallarioides (M = 49)

**Fig. 3:**
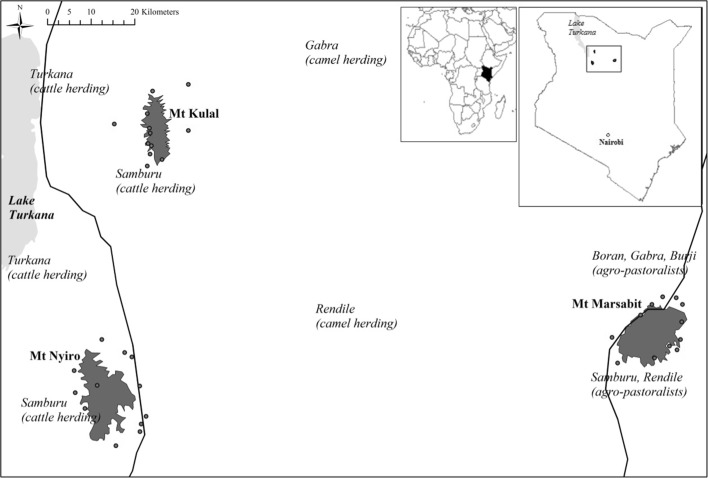
Selected mountains in northern Kenya and villages where focus-group discussions were organized with regard to main ethnic groups in the area. Black lines refer to major roads, dark grey areas to forests and red dots to villages studied

**Fig. 4:**
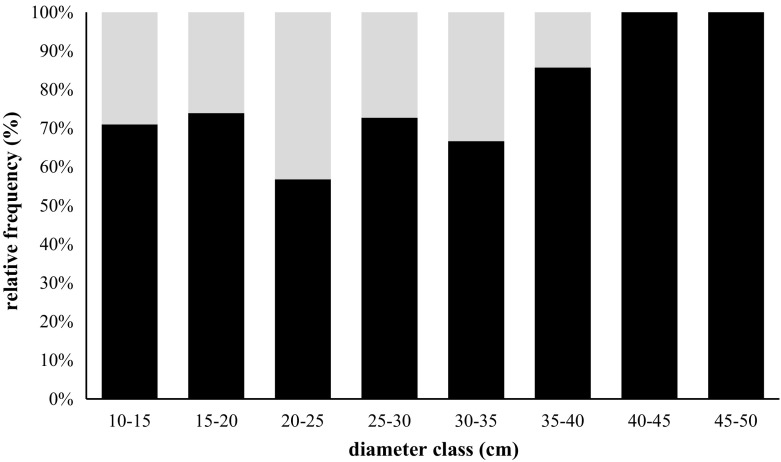
Percentage of severely pruned trees (black) and non-severely pruned trees (grey) of *Xymalos monospora* in Mt Nyiro

The population structure of most species studied followed a reverse J, with more individuals in smaller size classes than larger ones (Fig. 2). The exceptions were (1) *P. africana* and *O. capensis* in Mt Kulal and Mt Nyiro, for which some middle-size diameter classes had more individuals than smaller ones, and (2) *O. europaea* in Mt Marsabit, which had a bell-shaped SCD with no individuals in several small size classes (Fig. 2). SCD slopes were negative for most species, which indicates a good recruitment (Table 1). The exceptions were *P. africana* in Mt Nyiro (slope was close to 0) and *O. europaea* in Mt Marsabit, which had a flat slope.

### Forest carbon stocks

The differences in AGC between AGC pruned and AGC non-pruned ranged between 0.1 and 4.7% (Table 2). Mixed and elfin forests in Mt Nyiro, with numerous individuals of the abundant *X. monospora* tree being severely pruned, were the forest types with greater decrease in AGC (2.5 and 4.7% respectively, Table 2). These differences were not significant at *p* < 0.05.

**Table 2:** Above ground live tree carbon stocks (AGC) including severely pruned fodder trees (named AGC pruned), AGC if these trees had not been pruned (named AGC non-pruned), relative change in AGC; and fodder tree dominance (in terms of stem density) for the different types of forests studied

Mountain	Forest types	AGC pruned	AGC non-pruned	Change AGC (%)	Fodder trees amongst the dominant species
		(Mg C ha^−1^)	(Mg C ha^−1^)		
Mt Marsabit	Dry	54.9 ± 7.8	55.2 ± 7.7	0.6 ± 0.5	*Drypetes gerrardii*
	Mixed	94.9 ± 26.7	95.8 ± 26.1	1.1 ± 1.3	*Drypetes gerrardii, Olea capensis, Rinorea convallarioides*
Mt Kulal	Dry	73.6 ± 14.7	73.9 ± 15.1	0.4 ± 0.6	*Olea capensis*
	Mixed	145 ± 32.6	146.2 ± 33.4	0.7 ± 0.7	*Xymalos monospora, Pavetta gardeniifolia*
	Elfin	74.5 ± 0.8	74.5 ± 0.8	0.1 ± 0.1	*Xymalos monospora*
Mt Nyiro	Dry	113.2 ± 26.3	113.6 ± 26.4	0.3 ± 0.1	*Olea europaea, Olea capensis*
	Mixed	281.3 ± 62.7	287.5 ± 57.4	2.5 ± 3.7	*Xymalos monospora*
	Elfin	183.4 ± 82.7	191.9 ± 85.6	4.7 ± 1.5	*Xymalos monospora*

## Discussion

### Patterns of fodder harvesting

The main tree species used for fodder harvesting differed between mountains, despite Samburu pastoralists living around all three mountains, and all having more cows than goats and no sheep. Ethnicity is known to affect the choice of useful plant species and even plant parts of the same species (Assogbadjo et al. 2012; Sop et al. 2012). While species abundance and availability of alternatives affects the choice of preferred species for certain use (Jusu and Cuni-Sanchez 2014), our results also highlight that local preferences matter. Some of these local preferences can be explained by the harvesting techniques needed for fodder tree harvest (‘*it is easier to cut the stem of a small R. convallaroides tree than climb tall O. capensis to cut a few branches’*, participant comment in Mt Marsabit). In other cases local preferences are more difficult to explain: e.g. *X. monospora* and *P. africana* are relatively abundant in Mt Kulal and Samburu herders do not use it for fodder, despite Samburu herders in Mt Nyiro, 50 km away, doing so. *O. capensis, O. europaea* and *Maytenus undata* were also amongst the most important fodder trees in Ethiopia (Mekoya et al. 2008; Balehegn et al. 2015).

Fodder tree harvesting techniques differed between species. While for most species a few branches were cut, two species were often severely pruned: *X. monospora* in Mt Nyiro and *R. convallaroides* in Mt Marsabit (in the latter case main stem is cut). Interestingly, some *O. capensis* trees had their top branches unpruned. A similar pattern has been observed when Fulani herders harvest fodder tree of *Khaya senegalensis* (Desv.) A.Juss., an important fodder tree in West Africa (Gaoue and Ticktin 2007). Like for *K. senegalensis*, *O. capensis* were generally either totally pruned or else not harvested at all. This is likely because pruning trees is a dangerous activity (Petit 2003) that requires skill and experience. Large trees with tall and straight trunks are difficult to climb, and according to herders, only courageous and experienced male harvesters exploit them. For these large trees, harvesters maximize the amount of fodder harvested for each tree and thereby reduce the number of trees they need to climb.

With regard to size of the trees harvested, harvesters in our study did not prefer particularly large trees. This is different from other studies where harvesters tend to prefer larger trees: e.g. Gaoue and Ticktin (2007) for *Khaya senegalensis* or Shackleton et al. (2005) for a number of multipurpose tree species.

### Impacts on species’ populations

The population structure of two species studied (*O. europaea* in Mt Marsabit and *P. africana*) did not follow a reverse J, suggesting that fodder tree harvesting (combined with other uses) might be having a negative effect on these species’ populations. For *O. europaea* in Mt Marsabit, it is likely that it is the combination of fodder and firewood harvesting that is affecting this species. Although firewood harvesting has been strictly prohibited for a number of years in Masrabit national park, law has only been enforced since 2012, and some illegal extraction continues (personal observations). Greater law enforcement, increased community awareness about the need to sustainably manage canopy forest species and other conservation measures might be needed for this species (e.g. enrichment planting).

For *P. africana*, fodder harvesting might not be the problem, as *P. africana* populations were similar (with no individuals in several small size classes) in Mt Kulal and Mt Nyiro, despite this species having no use in Mt Kulal. A similar SCD has been reported from dry and intermediate montane forests in Mt Kenya (where its bark is commercialized as medicine, Nguta 2012), and in The Bale Mountains in Ethiopia (where it is a valuable timber species, Young et al. 2017). A review of different studies in Ethiopia showed that there is a need to clarify the natural reproductive strategy of this species (Young et al. 2017), as episodic recruitment might not be related to anthropogenic activities. This species might require special conditions for successful reproduction (e.g. a particularly wet year). Interestingly, local populations did not mention ‘declining populations’ for this species.

The population structure of the other six species studied followed a reverse J, suggesting that fodder tree harvesting (combined with other uses for some species) is not currently having a negative effect on these species’ populations. Presumably, such populations are relatively stable and, therefore, these species probably are not of urgent conservation concern in the forests studied, with the exception of *R. convallaroides*. *R. convallaroides* is often severely pruned, it does not sprout and local communities mentioned ‘declining populations’. If we had assessed smaller diameter classes (< 10 cm diameter), or seedlings and saplings, the population structure might have indicated a lack of regeneration. The same applies to *O. capensis*. More research is needed on these two species, so that their conservation status and management strategy can be clearly defined.

Overall, it should be highlighted that several factors (harvesting techniques, intrinsic characteristics of the species, other uses) should be considered while trying to relate species’ population structure to the impacts of wild harvesting. For instance, although *X. monospora* was severely pruned, fodder harvesting did not seem to negatively affect this species’ populations, as it is often multi-stemmed, and it re-sprouts easily. Other studies have highlighted the importance of considering intrinsic characteristics to the species when assessing the impacts of harvesting on plant populations (e.g. Schumann et al. 2011).

Our findings can also help suggest species for reforestation programs in montane forests. Instead of planting exotic species such as *Eucalyptus* spp., indigenous multipurpose trees could be used. Several studies have shown that indigenous trees are more useful to rural communities than exotic species (e.g. Faye et al. 2011), and can exhibit growth rates comparable to non-native species (e.g. Bare and Ashton 2016). Among the species we studied, *X. monospora* seems to be a good candidate, as it can withstand severe pruning. This species can be grown from seed, and although germination is slow, the germination rate is quite high (Mojeremane 2012). The potential of this species has already been highlighted by other authors, in view of its prospects and multiple uses, including timber, medicine and edible fruits (Mojeremane 2012).

### Impacts on forest carbon stocks

Fodder tree harvesting did not significantly affect forest carbon stocks, even in Mt Nyiro where > 70% of the dominant *X. monospora* trees were severely pruned. This result is related to the fact that *X. monospora* is a medium-sized understory tree and in this forest most carbon is stored in large trees. For example, in Mt Nyiro, *Podocarpus latifolius* (Thunb.) R.Br. ex Mirb. and *Faurea saligna* Harv. are the largest canopy trees, some of which being > 1 m diameter (personal observations.). Our finding that fodder tree harvesting did not significantly affect total forest carbon stocks is similar to that of Lung and Espira (2015) which reported that the removal of small stems for firewood does not significantly influence forest carbon stocks in Kakamega, a transitional moist forest in western Kenya.

### Implications of the findings and conclusions

Eight tree species were commonly harvested for fodder using different techniques (some branches, main stem, most branches except stem apex). Results indicate that fodder tree harvesting (together with other uses for some species) negatively affected one species (*O. europaea*), did not negatively affect four species, and more information is needed for three other species (*P. africana, O. capensis, R. convallaroides*). Harvesting techniques used, the intrinsic characteristics of the species, and the effect of other uses/factors should be considered when assessing the impacts of plant harvesting from the wild. Among the species we studied, *X. monospora* seems to be a good candidate for reforestation programs, as it can be easily propagated from seed, it is multipurpose and can withstand severe pruning (Mojeremane 2012).

With regard to the effects on carbon stocks, fodder tree harvesting did not significantly reduce forest carbon stocks. This suggests that local communities could continue to use these fodder trees even if a carbon project is established, which is particularly important as local communities, who are pastoralists, rely on these forests for their livelihoods (Cuni-Sanchez et al. 2016). Although our study is only a snapshot of current population structure of the studied species, it is a baseline which can be used to monitor changes in fodder harvesting and its impacts related to increasing droughts in northern Kenya and increasing human populations (especially around Mt Marsabit). Future studies could focus on quantifying how much tree fodder is harvested during each major drought event, and how this affects species’ population structure.

## Appendix

See Figs. 3 and 4.
